# Multisystem inflammatory syndrome (MIS-C) in Pakistani children: A description of the phenotypes and comparison with historical cohorts of children with Kawasaki disease and myocarditis

**DOI:** 10.1371/journal.pone.0253625

**Published:** 2021-06-21

**Authors:** Shazia S. Mohsin, Qalab Abbas, Devyani Chowdhary, Farah Khalid, Abdul Sattar Sheikh, Zuviya Ghazala Ali Khan, Nadeem Aslam, Omaima Anis Bhatti, Maha Inam, Ali Faisal Saleem, Adnan T. Bhutta

**Affiliations:** 1 Department of Pediatrics and Child Health, Aga Khan University, Karachi, Pakistan; 2 Cardiology Care for Children and Children’s Hospital of Philadelphia, Philadelphia, PA, United States of America; 3 National Institute of Cardiovascular Diseases, Karachi, Pakistan; 4 Aga Khan University Medical College, Karachi, Pakistan; 5 Department of Pediatrics, University of Maryland School of Medicine, Baltimore, MD, United States of America; Kaohsuing Medical University Hospital, TAIWAN

## Abstract

**Objectives:**

To determine clinical, laboratory features and outcomes of Multisystem Inflammatory Syndrome in children (MIS-C) and its comparison with historic Kawasaki Disease (KD) and Viral Myocarditis (VM) cohorts.

**Methods:**

All children (1 month– 18 years) who fulfilled the World Health Organization criteria of MIS-C presenting to two tertiary care centers in Karachi from May 2020 till August 31^st^ were included. KD and VM admitted to one of the study centers in the last five years prior to this pandemic, was compared to MIS-C.

**Results:**

Thirty children with median age of 24 (interquartile range (IQR)1–192) months met the criteria for MIS-C. Three phenotypes were identified, 12 patients (40%) with KD, ten (33%) VM and eight (26%) had features of TSS. Echocardiography showed coronary involvement in 10 (33%), and moderate to severe Left Ventricular dysfunction in 10 (33%) patients. Steroids and intravenous immunoglobulins (IVIG) were administered to 24 (80%) and 12 (41%) patients respectively while 7 (23%) received both. Overall, 20% children expired. During the last five years, 30 and 47 children were diagnosed with KD and VM, respectively. Their comparison with MIS-C group showed lymphopenia, thrombocytosis, and higher CRP as well as more frequent atypical presentation in MIS-C KD group with less coronary involvement. The MIS-C VM was more likely to present with fulminant myocarditis.

**Conclusions:**

Our MIS-C cohort is younger with higher mortality compared to previous reports. MIS-C is distinct from historic cohorts of KD and VM in both in clinical features and outcomes.

## Background

Severe acute respiratory syndrome coronavirus 2 (SARS-CoV-2) related disease (COVID-19) has been reported to have lesser and a milder disease presentation in children [1, 2]. However, in April,2020 there were reports from United States, Italy and other parts of the world describing a multi system inflammatory syndrome in children [3–5]. These reports described children presenting with a spectrum of clinical features with similarities to Kawasaki disease (KD), toxic shock syndrome (TSS) and viral myocarditis (VM). World Health Organization (WHO), Royal College of Pediatrics and Child Health (RCPCH) of United Kingdom and the Center for Disease Control (CDC) published criteria for diagnosed with MIS-C [6–8].

After the initial reports from USA and Europe, reports have also been published from Pakistan, India, and South Africa with almost similar spectrums of presentations of MIS-C [9–11]. The report of eight cases published from Lahore, Pakistan showed high incidence of coronary involvement (62.5%) while cardiac dysfunction was not a prominent feature in these patients. Karachi is the largest city of Pakistan and had the highest density of overall cases. As of 30^th^ November 2020, there have been 398,024 cases of COVID-19 in Pakistan, and children constitute around 10% of total cases [12]. The Aga Khan University Hospital (AKUH) and National Institute of Cardiovascular Disease (NICVD) are the two largest tertiary care pediatric centers in Karachi, Pakistan and serve as major referral centers for any complex patient with cardiac involvement or requiring pediatric intensive care unit (PICU) care. These institutions educated the primary care providers via live webinars and, social media about MIS-C and developed a common management algorithm for MIS-C patients (**S1 Appendix**).

In this study, we describe the clinical and laboratory features of children diagnosed with MIS-C at these sites with 2 months follow up and furthermore provide a comparison with 5-year historic data of patients diagnoses with KD and VM at AKUH. This comparison provides insights into the similarities and differences of MIS-C with KD and VM.

## Methods

Retrospective observational study was conducted at the two centers from 1^st^ April through 31^st^ August after approval from ethical review committee. The waiver of consent was granted for this study. (Reference # 2020-5715-14993). All children fulfilling the WHO’s MIS-C case definition, except for history of contact/exposure, were included. Children with a clear alternative etiological classification were excluded. We retrospectively reviewed data of patients diagnosed as KD and myocarditis at AKUH during the previous five years prior to the pandemic and compared the clinical and laboratory features of these children with MIS-C related KD and myocarditis. We also compared the laboratory parameters, disease spectrum of MIS-C and outcome of patients with and without known history of contact.

### Data collection and analysis

Data was extracted on a structured case report form from patients’ medical records. Data collection included demographic information (age, gender), clinical data (symptoms on presentation, clinical examination findings, relevant laboratory test result and echocardiographic details, SARS-CoV-2 polymerase chain reaction (PCR) test result, SARS-CoV-2 antibody test), management details (antibiotics, antivirals, immunoglobulin, steroids), need for PICU admission, their outcome and follow up. All patients were followed as per protocol (supplementary material) with latest follow-up till 15^th^ October 2020.

Based on their clinical presentation, patients were grouped into three main categories: a) KD like illness including KD and atypical KD definition according to AHA (labelled as MIS-C KD) [10]; and Non KD like illness which was subdivided into b) TSS (MIS-C TSS) and c) VM (MIS-C VM) as described in **Table 1**. These three groups were then compared using ANOVA (Analysis of Variance) for normally distributed variable, Kruskul Wallis test for non-normally distributed variables and Chi-square test for categorical variables. The MIS-C KD group were also compared to historic KD and MIS-C VM to Historic VM from last 5 years. Multiple comparison test was performed for those clinical or laboratory features whose overall significance were found p < 0.05 using Tukey test for normally distributed, Kruskul Wallis post hoc test for non-normally distributed variables. P-values are adjusted for multiple comparison using Bonferroni Method. Multinomial regression was also performed to predict the phenotype of disease form clinical and laboratory representation.

**Table 1:** Case definitions.

Variable	Definition
**Multisystem inflammatory Syndrome in children (MIS-C)**	MIS-C was defined as any patient fulfilling the criteria defined by WHO^α^ except for requirement for SARS-CoV-2 exposure contact history as that was hard to ascertain without the presence of widespread testing in the country.
**Kawasaki Disease (KD) Like illness**	Was diagnosed using the validated diagnostic criteria of the American Heart Association [13]. The group was subdivided in to KD and atypical KD. KD is diagnosed in the presence of fever for at least 5 d (the day of fever onset is taken to be the first day of fever) together with at least 4 of the 5 following principal clinical features. 1.Erythema and cracking of lips, strawberry tongue, and/or erythema of oral and pharyngeal mucosa. 2.Bilateral bulbar conjunctival injection without exudate. 3.Rash: maculopapular, diffuse erythroderma, or erythema multiforme-like. 4.Erythema and edema of the hands and feet in acute phase and/or periungual desquamation in sub-acute phase 5.Cervical lymphadenopathy (≥1.5 cm diameter), usually unilateral Atypical KD: Presence fever > 5days and 2–3 KD criteria With positive echocardiogram OR 3 or more Laboratory findings: 1. Anemia for age 2. Increased Platelets > 7 days of fever. 3. Albumin < 3.0 g/dl 4. Elevated AST^β^ ALT^∞^ 5. WBC^π^ > 15000 6. Pyuria.
**Coronary artery involvement**^**∞**^	Coronary involvement was categorized as only echo brightness without any ectasia or coronary dilation, and/or coronary aneurysm. Coronary dilation was defined as coronary artery diameter z-score of > + 2 to +3 while coronary artery diameter with a z score > +3 was labelled as severe ectasia or aneurysm. Echo brightness was described as appearance of bright broad echoes surrounding the coronary lumen extending for at least 1 cm along the artery, as compared to thin parallel echoes representing normal coronary artery walls distinct from the surrounding [14].
**The Non KD like illness**	**A. Viral Myocarditis (VM**)
	*Age related tachycardia, tachypnea, hypotension, elevated levels of pro–brain natriuretic peptide (proBNP) troponin (all during the first 24 hours of admission) and depressed LV^£^ function
	* Depressed LV function was defined as LV ejection fraction (LVEF) of < 50% and severe depressed as LVEF <30%.
	**B. Toxic Shock Syndrome (TSS**) like illness was defined was described as with signs of distributive shock, multi-organ injury and systemic inflammation. There will be age related tachypnea tachycardia hypotension, not responding to fluid boluses and requiring two inotropic support. The alternate diagnosis of other bacterial infection needs to be ruled [15].

^β^AST: aspartate aminotransferase

^∞^ALT: alanine transaminase

^£^LV: Left Ventricle

^π^WBC: White blood cell

^α^WHO: World Health Organization.

Data was entered and analyzed using STATA version 15.0. Results are presented as mean ± Standard deviation (SD) or median with interquartile range (IQR) and frequency with percentage. Appropriate statistical tests (Pearson chi-squared for categorical variable, two sample t-test for continuous normally distributed data and Wilcoxon rank sum test for non-normal data) were applied. A p value of <0.05 was taken as significant.

## Results

### Study population and characteristics

A total of 30 patients (77%) males) were included. Median age of the study population was 24 (IQR 9.5–60) months and 13 (44%) were under the age of 2 years (**Table 2**). The most consistent presenting symptom was fever (100%), with a median duration of 4 (IQR 3–5) days followed by Gastrointestinal symptoms (n = 21, 70%), rash (n = 16, 53%), hypotension (n = 14, 47%) and central nervous system symptoms like irritability, convulsions, and delirium (n = 11 (37%). Twelve patients (40%) had features of KD (MIS-C KD), 10 (33%) had myocardial dysfunction (MIS-C VM), and 8 (26%) had features of TSS (MIS-C TSS). The description of different clinical, laboratory and outcome features of three phenotype of MIS-C is presented in **Table 2**.

**Table 2:** Comparison of clinical and laboratory features of children presenting with multisystem inflammatory syndrome (MIS-C) (n = 30).

Characteristics	MIS-C KD^a^	MIS-C VM^b^	MIS-C TSS^c^	Total	

	N = 12	N = 10	N = 8	N = 30	P-value
Demographic Characteristics					
**Age in Month, Median (IQR)**	27 (9–60)	18(9.5–42)	30 (9.9–174)	24(9.5–60)	1.000
**Gender, n (%)**					1.000
Male	11 (92%)	6 (60%)	6 (75%)	23 (77%)	
Female	1 (8%)	4 (40%)	2 (25%)	7 (23%)	
**Symptoms on Admission**					
Fever	12 (100%)	10 (100%)	8 (100%)	30 (100%)	---
Length of Fever in Days, Median (IQR^d^)	5 (4–7)	4 (3–5)	5 (3–5)	4 (3–5)	1.000
Vomiting/Diarrhea	7 (58%)	6(60%)	8 (100%)	21 (70%)	1.000
Delirium, irritability, or convulsions	4 (33%)	4 (40%)	3 (38%)	11 (37%)	1.000
**COVID-19 PCR** ^**e**^**Test Done**	12(100%)	6(60%)	8(100%)	26(86.67%)	1.000
Positive	4 (33%)	5 (83%)	5 (63%)	14 (54%)	
Negative	8 (67%)	1 (17%)	3 (38%)	12 (46%)	
**COVID-19 antibody Test Done**	5 (41%)	0 (0%)	2 (25%)	7(23%)	0.09
Positive	5 (100%)	0(0%)	2 (100%)	7 (100%)	
Negative	0 (0%)	0(0%)	0(0%)	0 (0%)	
Leucocyte, Median (IQR) (×10^9^/L)	11(9–14)	9.05 (6.7–12.2)	15.5 (10–19)	11 (9–16)	1.000
Neutrophils, Median (IQR) (%)	75 (68–81)	73 (64–75)	86 (66–87)	75 (65–85)	1.000
Lymphocytes, Median (IQR) (%)	17 (12–22)	20 (15–30)	11 (10–26)	18 (11–24)	1.000
Platelets Count, Median (IQR) (×10^9^/L)	304 (245–389)	225 (129–335)	152 (96–224)	230 (140–330)	0.300
CRP^f^, Median (IQR) (mg/L)	67.91 (25–167)	26(1.10–61)	19.50 (6.7–93.7)	31.00 (3.05–84.85)	1.000
D-Dimer Median (IQR) (ng/ml)	30 (2.3–99)	15 (0.20–30.00)	12.00 (1.40–18.00)	3.6(2.1–9.6)	1.000
Ferritin, Median (IQR) (ug/L)	567 (151–1491)	224(99–656)	673(316–1480)	587 (276–1111)	1.000
LDHh, Median (IQR) (IU/L)	575 (333–1933)	1650 (460–980)	408(208–830)	617 (372–1696)	1.000
LVEF^i^ on Admission, Median (IQR) (%)	57 (46–61)	30 (25–45)	40(39–57)	46 (30–61)	1.000
**Coronary Involvement n (%)**	7 (58%)	0 (0%)	3 (38%)	10 (33%)	1.000
Dilation	1 (14%)	0 (0%)	1 (33%)	2 (20%)	
Aneurysm	2 (29%)	0 (0%)	0 (0%)	2 (20%)	
Echo bright	4 (57%)	0 (0%)	2 (67%)	6 (60%)	
**Treatment**					
PICU ^j^ Admission	10 (83%)	9 (90%)	8 (100%)	27(88%)	1.000
Mechanical Ventilation	0 (0%)	9(90%)	4(50%)	12(40%)	0.675
Inotrope Support	4(33%)	10(100%)	8(100%)	22(73%)	1.000
Steroids	7 (58%)	9 (90%)	8 (100%)	24 (80%)	0.964
IVIG^k^	10 (83%)	0 (0%)	2 (25%)	12 (41%)	**<0.001**
Both Steroids and IVIG	5 (42%)	0 (0%)	2 (25%)	7 (23%)	1.000
Anticoagulation	12 (100%)	6 (60%)	5 (63%)	23 (77%)	0.705
**Follow-Up**					**0.1.35**
Yes	12 (100%)	3 (43%)	6 (86%)	21 (81%)	
No	0 (0%)	2 (57%)	1 (14%)	5 (19%)	
**Outcome**					**0.180**
Survived	12 (100%)	5 (50%)	7 (88%)	24 (80%)	
Expired	0 (0%)	5 (50%)	1 (13%)	6 (20%)	

KD = Kawasaki disease

^b^VM = viral myocarditis

^c^TSS = Toxic shock syndrome

^d^IQR = Interquartile range, ^e^PCR = Polymerase chain reaction

^f^CRP = c-reactive protein

^h^LDH = lactate dehydrogenase

^i^LVEF = left ventricular ejection fraction

^j^PICU = pediatric intensive care unit

^k^ IVIG = intravenous immunoglobulin showed, α = statistical difference was found in group MIS-C KD after performing pair comparison Kruskul Wallis test

**Fig 1:**
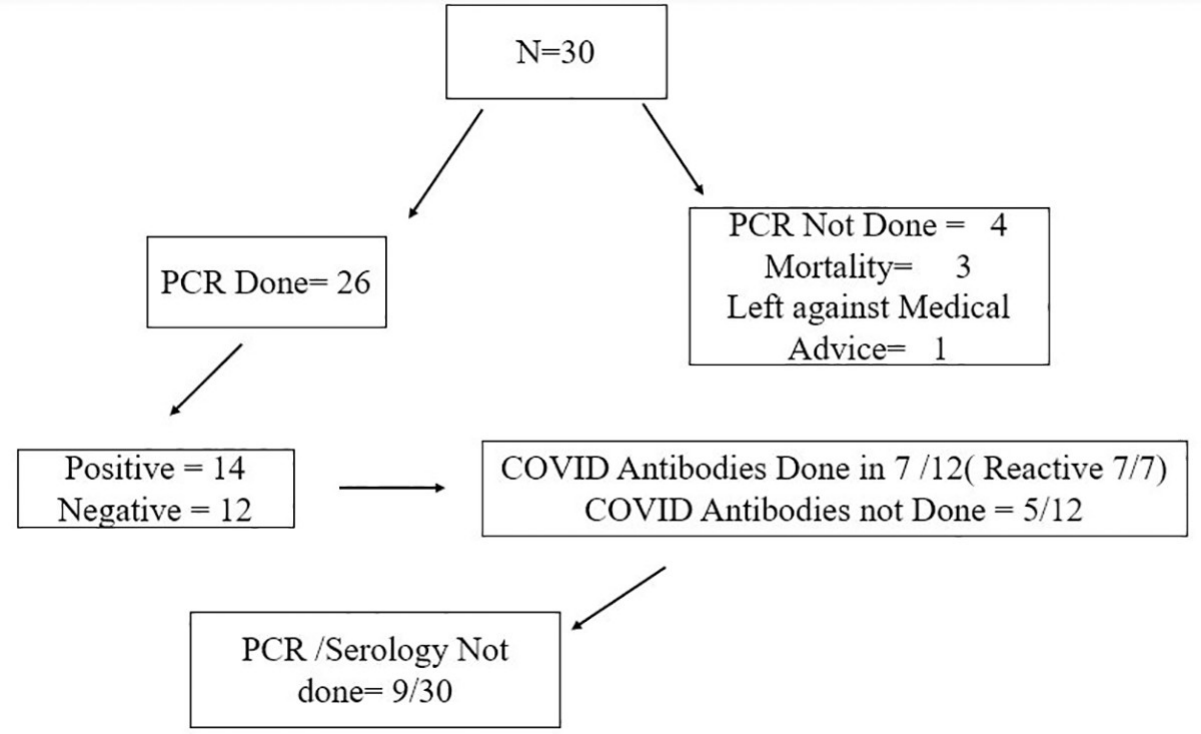
SARS CoV PCR and serology results.

Eighty six percent of patients (26/30) were tested using SARS-CoV-2 PCR and 14/26 (53.8%) were positive. COVID-19 serology was done in remaining 7/12 (23%) and all were reactive. Serology was not performed in those with positive PCR due to cost issues. Four patients in our cohort unfortunately did not get either PCR or antibody but were included based on clinical suspicion and history of contact **(Fig 1)**. Positive contact history for COVID-19 was present in 21/30 (70%) patients. There was no difference between symptoms at presentation, demographics, laboratory parameters and outcomes between patient with or without a history of contact (**Table 1 in S2 Appendix**).

### Clinical phenotypes

There was total 12 patients with MIS-C KD like illness during the pandemic. Distinct laboratory features are described in **Table 2**. Nine (75%) patients were diagnosed as atypical KD and 3 (25%) were diagnosed as KD. Coronary involvement was seen in 7/12 (58.3%) patients. Four (57%) had perivascular brightness, two had coronary dilation and one had severe coronary aneurysm formation. IVIG was administered in 10/12 (83%) patients and all 12 patients survived. Complete coronary resolution was seen in 6/7 (85%) patients on a median follow up of 1 month. Ten patients (33%) presented with MIS-C VM and 8(26%) with MIS-C TSS **(Table 2)**. On comparing the 3 phenotypes, only significant difference after Bonferroni correction, was use of IVIG in MISC-KD group. The p-value of 0.045 in Trop-I levels in MISC-VM and < 0.001 in presence of Rash in MISC-KD group was part of operational definition so were not considered significant. Due to small sample size, we could not construct a multi-variate model to predict phenotype from the clinical and laboratory data **(Table 4 in S2 Appendix)**.

### Management and outcome data

Overall, 26/30 (80%) patients were admitted to the PICU, 22(73%) required inotropic support and 20(40%) mechanical ventilation. Intravenous steroids were given to 24 (80%) patients and intravenous immunoglobulins (IVIG) was administered to 12 (41%) patients while 7 (23%) received both IVIG and steroids. Anticoagulation was prescribed to 23 patients (77%). Overall mortality was 20% (6/30). These 6 patients are described individually in **Table 3 in S2 Appendix**.

There was 100% survival and follow up in the MIS-C KD group. IVIG was administered in 10 (83%) patients. They were kept on low dose Aspirin and followed-up with a transthoracic echocardiogram, with 85% (6/7) resolution of coronary involvement.

The mortality was 33% (6/18) in MIS-C non KD cohort. Majority 5/6 (83%) belonged to MIS-C VM group and one patient had suspicion of TSS. All survivors were prescribed oral prednisolone on discharge along with low dose Aspirin. Seventy five percent (9) of these followed their primary cardiologist and reported to have resolution of symptoms, laboratory markers and echocardiographic features within one month of onset of symptoms.

### Comparison of historic KD and VM with MIS-C KD and VM

**Fig 2:**
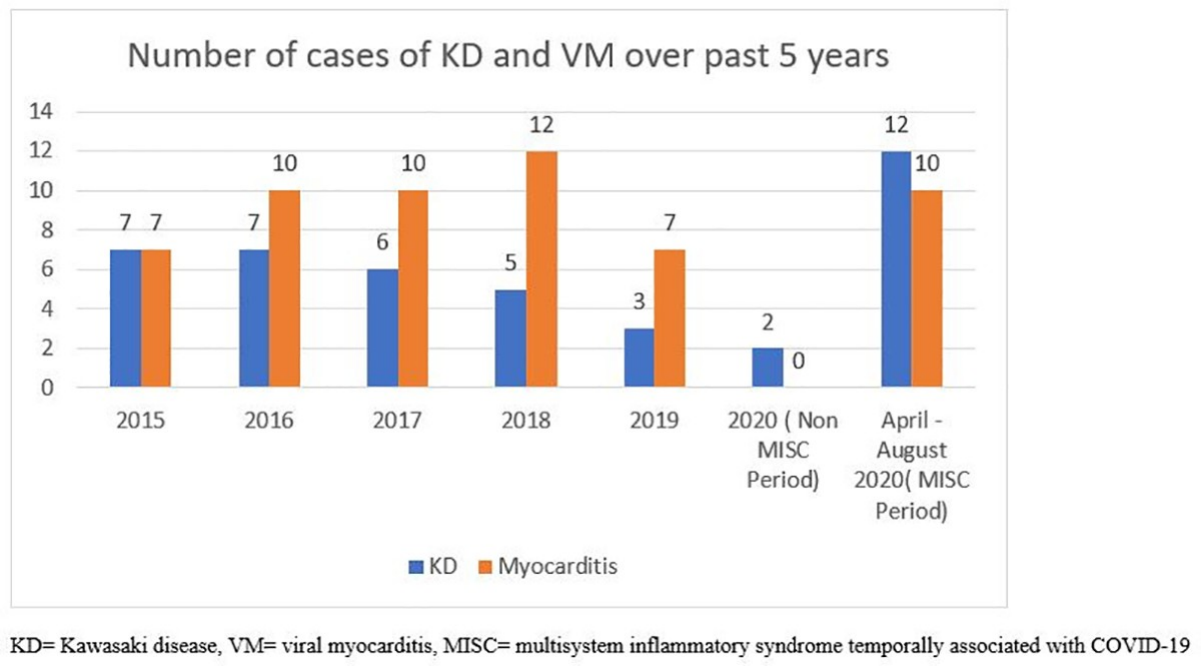
Number of cases with Kawasaki Disease (KD) and Viral Myocarditis (VM) over past 5 years.

**Fig 3:**
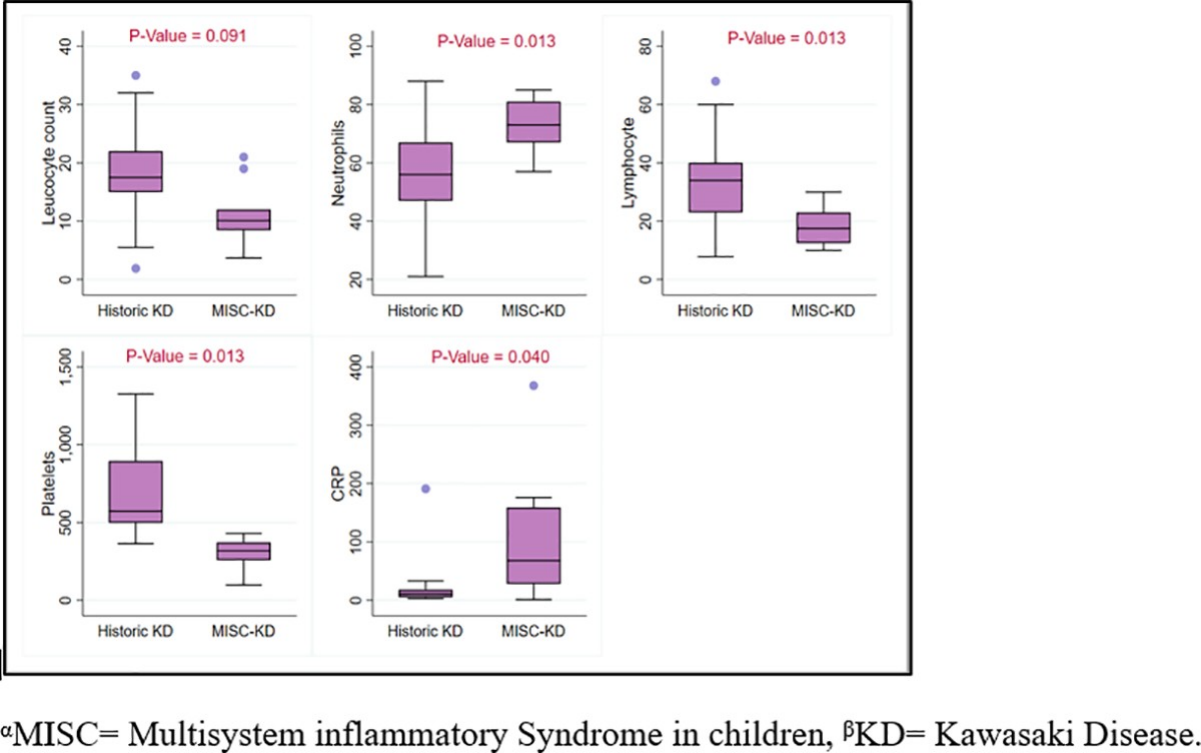
Laboratory features comparison in MISC and historic KD.

During the last 5 years; 30 patients were diagnosed with KD at the AKUH (0.48 cases per month). From March till July 2020; 12 patients were diagnosed with MIS-C KD (2.4 cases per month) which constitutes a 5-fold rise in cases (**Fig 2**). Comparison of two groups showed significant difference in presentation (**Table 3**). There was no gender predominance,75% (9/12) presented as atypical KD as compared to only 20% (6/30) in historic KD (p value = <0.013). The median lymphocyte count (17 vs 34 x10^9^/L, p = <0.091) and median platelet count (304 vs 573 x10^9^/L, p = <0.013) were lower in MIS-C KD group when compared to the historic KD group. The median C-Reactive Protein was higher in the MIS-C KD cohort (68vs 11 mg/L; p = 0.040) as well (**Fig 3**). In the MIS-C group, 58.3% had coronary involvement, though mainly (57%) perivascular brightness and majority (86%) resolved. In the historic KD cohort, main coronary involvement was coronary dilation (50%) followed by aneurysm (33%) and perivascular brightness (17%), with 56% resolution rate. The median left ventricular ejection Fraction (LVEF) in MIS-C KD was57%(IQR = 46–61) vs 67% (IQR = 63–72) in historic KD (p value = <0.001).

**Table 3:** Comparison of clinical and laboratory characteristics of historic and MIS-C Kawasaki disease.

Characteristics	Historic KD	MIS-C KD	Total	P-value
	N = 30	N = 12	N = 42	
**Demographic Characteristics**				
**Gender, n (%)**				**0.390**
Male	17 (57%)	11 (92%)	28 (67%)	
Female	13 (43%)	1 (8%)	14 (33%)	
**Clinical Findings**				
**Diagnosis, n (%)**				**0.013**
KD^a^	24 (80%)	3 (25%)	27 (64%)	
Atypical KD	6 (21%)	9 (75%)	15 (36%)	
**Coronary Type, n (%)**	18(60%)	7(58%)	25(59%)	1.000
Dilated	9 (50%)	2 (29%)	11 (46%)	
Aneurysm	6(33%)	1 (14%)	7(28%)	
Echo brightness	3 (17%)	4 (57%)	7 (28%)	
**Laboratory Findings**				
Leucocyte count, Median (IQR^b^) (×10^9^/L)	18 (15–22)	11(9–14)	16 (9–21)	**0.091**
Neutrophils, Median (IQR^c^) (×10^9^/L)	56 (47–67)	75 (68–81)	66 (52–73)	**0.013**
Lymphocyte, Median (IQR) (×10^9^/L)	34 (23–40)	17 (12–22)	28 (18–36)	**0.013**
Platelets Count, Median (IQR) (×10^9^/L)	573 (498–895)	304 (245–389)	529 (368–682)	**0.013**
LVEF, Median (IQR) (%)	67 (63–72)	57 (46–61)	65 (60–72)	**<0.001**
CRP, Median (IQR) (mg/L)	11 (5–18)	68 (25–167)	13 (5–33)	**0.039**
**Treatment / Dosage**				
**IVIG**^**c**^**, n (%)**				**0.260**
Yes	30 (100%)	10 (83%)	40 (95%)	
No	0 (0%)	2 (17%)	2 (5%)	
**Coronary involvement Outcome\Follow-up, n (%)**	n = 18	n = 7	n = 25	1.000
Resolved	10 (56%)	6 (86%)	16 (64%)	
Not resolved	3(13%)	1(14%)	4 (16%)	
No Follow up	5 (29%)	0(0%).	5(25%)	

^a^KD = Kawasaki disease

^b^IQR = Interquartile range

^c^IVIG = Intravenous immunoglobulin.

The previous five-year data of historic VM showed a total of 47 patients. The frequency was 0.78 cases per month as compared to 1.66 cases per month during the pandemic period, which constitutes a 2.1-fold increase., Pericardial effusion was more likely to be seen in MIS-C VM (78% vs 0% p value = <0.001) and reversal of LVEF to normal was more likely in MIS-C VM survivors (100%vs 21.4% p value = 0.04). Whereas 51% of historic VM cohort still developed dilated cardiomyopathy (DCMP), none of MIS-C VM developed DCMP (p value = 0.00 (**Table 4**).

**Table 4:** Comparison of clinical and laboratory characteristics of historic and MIS-C viral myocarditis.

Characteristics	Historic Myocarditis	MISC-Myocarditis	Total	p-value
	N = 46	N = 10	N = 56	
**Laboratory Findings**				
Leucocyte count, Median (×109/L) IQR	14 (12–20)	9 (7–11)	14 (11–19)	**0.169**
Neutrophils Median (×109/L) IQR	49 (36–62)	73 (64–75)	51 (38–72)	**0.040**
Lymphocytes Median (×109/L) IQR^β^	44 (30–56)	20 (15–30)	39 (20–53)	**0.040**
Platelets Median (×109/L) IQR	400 (300–515)	225 (129–335)	382 (249–478)	**0.040**
**Pericardial Effusion**				**<0.001**
Present	0 (0%)	7 (78%)	7 (13%)	
Not Present	46 (100%)	2 (22%)	48 (87%)	
**Outcome**				
Survived	33 (71%)	5 (50%)	38 (67%)	**0.130**
Normal Left ventricle Function	10 (30%)	5 (100%)	15 (39%)	**0.040**
Dilated Cardiomyopathy	23 (69%)	0 (0%)	23 (60%)	**<0.001**

^α^MISC = Multisystem inflammatory Syndrome in children

^β^IQR = Interquartile range.

## Discussion

SARS-CoV-2 infection in children and adolescents can cause MIS-C, which shares clinical similarities with KD, TSS and VM. We describe 30 hospitalized children with MIS-C diagnosed in first five months after the pandemic hit the city of Karachi Pakistan, in March 2020. This constitutes one of the largest cohort of patients reported from a low-or-middle income country (LMIC). Our cohort of patients is younger and had a higher mortality rate compared to published reports, but the presenting symptoms and range of organ involvement was like that reported by others [3–5, 16–20].

The diagnosis of MIS-C was made in most patients based on a positive PCR or serology for SARS-CoV-2. In nine patients in our cohort with either a negative PCR or serology or no COVID testing performed, we made the diagnosis of MIS-C after the exclusion of other conditions like bacterial and viral infections and with suspicion of exposure. We also compared demographics, clinical feature, biochemical parameters, SARS-CoV-2 PCR results, and outcomes of patients fulfilling WHO definition with history of contact and those with no history of contact and found no difference between the groups. However, serology results were significantly more likely to be positive in the group with positive history of contact. This highlights the fact that in LMIC with high rates of community transmission and increased number of asymptomatic (or mildly symptomatic) patients coupled with limited laboratory resources and access to testing, history of exposure may not always be able to be ascertained. In the face of wide community spread, history of contact may have less significance. Similar concern was conveyed in some correspondence from South Africa [11].

### MIS-C-KD like illness phenotype

KD is an autoimmune disease, seen in children < 5 years of age and hallmark is coronary artery dilation and aneurysm formation. During the SARS-CoV-2 pandemic, we report a 5-fold increase in MIS-C related KD-like illness compared to the prior five years (**Fig 1**). A 30-fold increase in KD-like illness was reported from Italy during the SARS CoV-2 pandemic, when compared to previous 5 years [16]. Ouladali et al from France reported an increase in KD during SARS-CoV-2 and previously with influenza A H1N1 pandemic in 2009, thus suggesting a role of viral infections as triggers for KD (21). We showed that MIS-C KD can be considered a *distinct* entity, different from Historic KD. MIS-C KD differs from prior KD in its presenting features (male predominance and higher percentage of atypical KD), laboratory markers (lower lymphocyte and platelet counts and higher CRP), coronary artery involvement pattern and outcome (more likely to have perivascular brightness followed by complete resolution within 1 month). These features may help in differentiating MIS-C KD from KD in an LMIC and identify patients in which other laboratory investigations should be offered, as also shown in previously published data [10, 16]. Our cohort of MIS-C KD had a mild course and showed good response to IVIG, with complete resolution of laboratory and echocardiographic abnormalities at 6 weeks of follow up. These results are like the ones reported from India but different from France, where the course was more severe [10, 20, 21].

### MIS-C VM and TSS phenotype

MIS-C VM has now been well described in the literature. MIS-C VM may be due to direct myocardial damage or delayed host response as in KD. Clinical presentation can be varied, ranging from mild symptoms to fulminant myocarditis [22, 23]. Our cohort showed fulminant myocarditis, with 50%mortality, and complete recovery in survivors. Children with MIS-C VM were treated primarily with steroids as limited supply and additional cost precluded treatment with IVIG. Also, none of the children received Tocilizumab and Anakinra or Extracorporeal support with plasma exchange, or ventricular assist device, which are available and frequently used in high income countries. The increased mortality in our patients with depressed LVEF when compared to other phenotypes from the same cohort and other published cases, could be multifactorial, including but not limited to delays in accessing care, delayed referrals to facilities with pediatric cardiology and critical care services, and limited availability of IVIG and other advance treatment options. Furthermore, if advanced treatments were to become available in resource-limited environments, they would most benefit children with MISC-VM.

We also compared MIS-C VM to Historic VM from previous five years and found remarkable difference in presentation and outcome of MIS-C VM. The presentation was more likely to be fulminant in children with MIS-C VM with decreased LVEF, severe valvar regurgitation and presence of pericardial effusion. Similar findings have been reported by Grimaud et al. [24] Though the mortality was high (50%), 100% of the survivors had recovered their LV function, valve regurgitation and pericardial effusion to normal within one month. In comparison, the historic VM cohort showed 71% survivors, with progression of 70% survivors to DCMP with persistent LV dysfunction, and complete recovery in remaining 30%. Our comparison emphasizes that MIS-C VM behaves more like fulminant VM with increased mortality but complete recovery, if treated promptly. Based on these results, we suggest early, aggressive management of children with MIS-C VM, as reported by others as well [18, 25]. The mortalities in our MIS-C VM cohort were within first 6 hours of presentation. The clinical presentation, along with distinct laboratory and echocardiographic features can help make the diagnosis of MIS-C VM and timely management could limit mortality in this group [26].

Eight (26%) of our patients presented with symptoms consistent with TSS in the setting of MIS-C. Their differentiating features from other subtypes were presence of vomiting, diarrhea, fluid refractory shock, requiring inotropic support and muco-cutaneous changes and preserved cardiac functions. This sub type was different from myocarditis both in presentation and outcome. More frequent coronary involvement (3 vs 0) and mild LV dysfunction with a median EF of 48% vs 30% was seen in MIS-C TSS when compared to MIS-C VM. All patients in this group received steroids and inotropes, and 25% received both IVIG and steroids. There was one mortality in this group, which was after 48 hours of admission. Due to a gap in our coding system, we could not compare the MIS-C TSS group to a historic cohort of similar patients like in other subgroups.

It is possible that the three phenotypes of MIS-C that we describe (KD, VM and TSS) represent various stages in the continuum of the evolving pathophysiology of MIS-C. It is hard to ascertain the onset of infection with SARS-CoV-2 in most of our patients and the natural history of MIS-C is still to be fully understood.

We have been able to follow up 21 of the 24 (87.5%) patients who survived to discharge. All survivors in our cohort received steroids while only 2/6 (40%) of the expired received steroids. Results from the RECOVERY trail in adult patients hospitalized with COVID-19 requiring respiratory support who received dexamethasone administration for 10 days showed a reduction in 28-day mortality [27]. Extrapolating this to MIS-C, we can say that steroids likely help to modulate the hyper inflammatory and prothrombotic responses seen in severe COVID-19 disease. Further investigation into the role of steroids in MIS-C need to be performed.

These results are encouraging and suggest that early diagnosis and treatment can be lifesaving. However, long-term follow up is required to perform surveillance for any late sequalae of this illness.

### Limitation of study

Our study is a report from two tertiary-care centers and does not constitute a city-wide or a country-wide report. While aggregate data for COVID-19 cases does exist, no national or local surveillance system to detect MIS-C currently exists in Pakistan [12]. Thus, no inference can be made on rates of illness. Four patients (13%) patients could not get either PCR or serology and the diagnosis of MIS-C was based on known contact with COVID-19 patients and their laboratory features, 3 of these could not survive. Furthermore, we did not collect information on diastolic dysfunction and strain assessment in echocardiographic assessment of the patients. Given the quite high mortality and, the centers being referral centers, patients may have been late in presentation or getting to the institutions. Another limitation was that classification of MI-C phenotypes has not been validated due to small sample size, in future data registries for MISC may help validating this classification.

## Conclusion

We report a large cohort of cases with MIS-C from Pakistan. Our data suggest that mortality from VM-like MIS-C, probably can be more in LMIC compared to high income countries. Our work demonstrates that COVID-19 and MIS-C are not benign conditions in children. This is especially true when cost limits treatment. We have shown three distinct yet overlapping phenotypes of MIS-C (KD, VM and TSS like presentations). In comparison to historic cohorts, MIS-C KD and VM can be labeled as distinct entities based on their clinical, laboratory and echocardiographic features.

## Supporting information

S1 Appendix A suggested management algorithm for MIS-C. (DOCX)

S2 Appendix Table 1. Clinical and laboratory features and outcome of patients presenting with MIS-C according to WHO case definition with or without history of contact. Table 2. Normal range of different laboratory values. Table 3. Expired Patients (n = 6) during the study period. Table 4. Multinomial model to predict phenotype of disease from clinical and laboratory data.
(DOCX)
