# Supplementary material for: Multisystem inflammatory syndrome (MIS-C) in Pakistani children: A description of the phenotypes and comparison with historical cohorts of children with Kawasaki disease and myocarditis
Source: PLoS One. 2021 Jun 21;16(6):e0253625. doi: 10.1371/journal.pone.0253625 (PMC8216534; doi:10.1371/journal.pone.0253625)
Supplement: S1 Appendix — (DOCX) [file pone.0253625.s001.docx]

**S1 Appendix**

| **BACKGROUND:**  Recently, a possible link has been reported between a pediatric multi-system inflammatory syndrome (PMIS) temporally associated with COVID-19 in Europe and the U.S., including over 100 cases in New York State in people <19 years of age. Patients are presenting with fever, laboratory evidence of inflammation and single OR multi-organ dysfunction.  Clinical features of this syndrome resemble those seen in other diseases including Kawasaki’s Disease (KD), Toxic Shock Syndrome (TSS) and Macrophage Activation Syndrome (MAS)/Hemophagocytic Lymph histiocytosis Syndrome (HLH). It is unknown whether this syndrome is specific to children, or if it may also be seen in adults. These guidelines are designed to maximize diagnostic opportunity, provide a streamlined approach to initial management, and provide some guidance for ongoing therapy and evaluation, in resource limited LMIC.  **GUIDELINES:**  These guidelines are intended as a general guide and should be applied and interpreted with caution and are likely to change over time. Departure from these guidelines may be appropriate and necessary in certain clinical circumstances (guidelines as of 6/8/2020).  **PURPOSE:**  To aid in the work-up, management and follow up of pediatric patients (< 19-year-old) with confirmed or suspected COVID-19 multi-system inflammatory syndrome (CMIS). These guidelines are not for the management of primary (active) COVID-19 infection. |
| --- |
| \| **CASE DEFINITION: BY WHO on 15-MAY-2020** \| \| --- \| \| Children and adolescents 0–19 years of age with fever > 3 days \| \| AND two of the following: \| \| a) Rash or bilateral non-purulent conjunctivitis or muco-cutaneous inflammation signs (oral, hands or feet). \| \| b) Hypotension or shock. \| \| c) Features of myocardial dysfunction, pericarditis, valvulitis, or coronary abnormalities (including ECHO findings or elevated Troponin/NT-proBNP), \| \| d) Evidence of coagulopathy (by Prothrombin time ( PT) elevated d-Dimers). \| \| e) Acute gastrointestinal problems (diarrhea, vomiting, or abdominal pain). \| \| AND Elevated markers of inflammation such as ESR, C-reactive protein (CRP), or procalcitonin. \| \| AND No other obvious microbial cause of inflammation, including bacterial sepsis, staphylococcal or streptococcal shock syndromes. \| \| AND Evidence of COVID-19 (RT-PCR, antigen test or serology positive), or likely contact with patients with COVID-19. \| |
| \| - **INITIAL LAB AND IMAGING WORK-UP** - **Essential labs**: - Complete blood picture with differentials (Lymphopenia, Neutrophilia), - CRP - Chest Xray - Blood Culture and sensitivity,   **Recommended Labs**: To aid in diagnosis of cytokine storm and treatment options. These might not be possible in all centers.   - Ferritin, - Procalcitonin, \| - D-dimer, - PT, - Lactic acid dehydrogenase (LDH), - Dengue IgM antibodies - **Suspected organ Involvement labs**: - Alanine transaminase (ALT) - Blood urea nitrogen and creatinine ratio ( BUN:Cr) - TROP I Pro brain natriuretic peptide (BNP) \| \| --- \| --- \| \| - COVID PCR = is not required for diagnosis but should be sent - Serology (Before starting IVIG)—For Diagnosis \| \| \| **ECHOCARDIOGRAM:**   - Mitral valvular regurgitation - Left ventricular function - Coronary artery dilation - Perivascular brightness - Pericardial Effusion - Tricuspid regurgitation. - Pulmonary regurgitation   **A NORMAL ECHO DOES NOT RULE OUT THE DIAGNOSIS, CHANGES IN CARDIAC FUNCTION MAY OCCUR RAPIDLY** \| \| \| **Other Supportive Labs according to Organ involvement:**   - Head imaging if focal deficit, seizure, meningeal signs. - Lumbar puncture if clinical sx/sx of meningitis/encephalitis (opening pressure, cell count, glucose, protein, culture; additional studies per ID recommendations) - stool for bacterial studies in patients presenting with diarrhea \| \| |
| \| **Clinical Features**  **A. Like Kawasaki Disease (KD)** \| **A-Classic KD** is diagnosed in the presence of fever for at least 5 d (the day of fever onset is taken to be the first day of fever) together with at least 4 of the 5 following principal clinical features.   1. Erythema and cracking of lips, strawberry tongue, and/or erythema of oral and pharyngeal mucosa. 2. Bilateral bulbar conjunctival injection without exudate. 3. Rash: maculopapular, diffuse erythroderma, or erythema multiforme-like. 4. Erythema and edema of the hands and feet in acute phase and/or periungual desquamation in subacute phase 5. Cervical lymphadenopathy (≥1.5 cm diameter), usually unilateral   **B-Atypical KD.**   - Presence fever > 5days and 2-3 KD criteria - With positive echocardiogram OR   3 OR MORE Lab finding:   1. Anemia for age 2. Increased Platelets > 7 days of fever. 3. Albumin < 3.0 g/dl 4. Elevated ALT 5. White blood cell count > 15000 6. Pyuria   Coronary involvement was also categorized as   1. Only echo brightness without any ectasia, dilation, aneurysm 2. Coronary dilation with a z-score > + 2 to 3. 3. Severe ectasia, aneurysm or zscore > +3.   Echo brightness was described as appearance of bright broad echoes surrounding the coronary lumen extending for at least 1 cm along the artery, as compared to thin parallel echoes representing normal coronary artery walls distinct from the surrounding \| \| --- \| --- \| \| 1. **MIS-C Toxic Shock Syndrome (TSS)**   With Refractory vasodilatory shock- (vasoplegic Similar to septic shock)  . \| - Patients with signs of distributive shock, multi-organ injury and systemic inflammation. There will be age related tachypnea tachycardia hypotension, not responding to fluid boluses and requiring two inotrope support. The alternate diagnosis of other bacterial infection needs to be ruled out \| \| 1. **MISC- C with Viral Myocarditis (VM)**   1-Myocardial dysfunction like myocarditis.  2-Isolated arrythmias without cardiac dysfunction \| - Age related tachycardia, tachypnea, hypotension, elevated levels of pro–brain natriuretic peptide (proBNP) troponin (all during the first 24 hours of admission) and depressed LV function   Depressed LV function was defined as LV ejection fraction (LVEF) of < 50% and severe depressed as LVEF <30 %.   - Arrythmias can be any type, and there can be block and Long QT as well. \| |
| - **Therapeutic Interventions **if clinical suspicion is high for MIS-C, do not wait for coronavirus testing results to initiate therapy****  1. **Supportive care.** 2. **Resuscitation**  \| Fluids \| 10cc/kg aliquots of Normal Saline if evidence of dehydration/shock; carefully assess response/tolerance of fluid (hemodynamic response, lung exam/liver edge; bedside ultrasound exam of Inferior vena cave if available) as boluses are administered \| \| --- \| --- \| \| Vasoactive \| Shock with poor perfusion:  Epinephrine, 0.02-0.05 mcg/kg/min, titrate to effect; use with caution in patients with extreme tachycardia.  Consider addition of milrinone, 0.25 – 0.5 mcg/kg/min if oxygen delivery remains inadequate \| \| Shock with normal/hyper perfusion:  Norepinephrine, 0.02-0.05 mcg/kg/min, titrate to effect; use with caution in the setting of myocardial dysfunction.  Consider vasopressin, 0.1-2 mcg/kg/min if hypotension is refractory tocatecholamine infusions \| |
| 1. **Immunomodulators:** 2. Intravenous Immunoglobulins (IVIG=2 gm /kg, max 160g; For obese patients (BMI>30), use adjusted body weight for dosing, which is [(ideal body weight + 0.4 x (actual body weight - ideal body weight)]   Notes: Dose in two aliquots if needed for fluid sparing. Watch for fluid overload, cardiac decompensation. Can repeat in 24-48 hours if not improved.  Consider 2^nd^ dose in refractory cases of KD: 1. If fever persists. 2. CRP or ESR not improving. 3. Coronary dilation.  **#Methylprednisolone**   \| Mild Clinical severity \| Minimal Organ involvement, no need for respiratory or inotrope support \| Methylprednisolone^#^  2mg/kg/day \| Steroid Taper  2-3 weeks \| \| --- \| --- \| --- \| --- \| \| Moderate clinical severity \| Oxygen requirement, Mild to moderate organ involvement, need of inotropes \| 10mg/kg x1, then 2mg/kg/day \| 4-6 weeks \| \| Severe clinical severity \| Need for non-invasive or invasive ventilatory support, moderate or severe organ injury including moderate to severe ventricular dysfunction \| 20-30mg/kg/day for 1-3 days, then 2mg/kg/day \| 6-8 weeks \|   ^x^ = Slow tapering over a period of 4-8 weeks monitoring clinical and lab markers of inflammation like CRP or ESR. is recommended.  #= Methylprednisolone to be used once clinical judgement has ruled out Leukemia, otherwise consult pediatric oncologist or experts  @ Tocilizumab in Selected or refractory cases after speaking to experts (Pediatric cardiologist, intensivist or rheumatologist) |
| - Anticoagulation: Aspirin 50 mg /kg /day if coronary involvement. (Till fever is there or CRP start coming down) - Enoxaparin: [referred when D-Dimer is high, later low dose aspirin. (Consider full clinical picture when deciding anticoagulation regimen - Consider for patients who fulfil criteria for Kawasaki’s disease; may initiate at moderate to high dose (particularly if coronary arteries changes by echo) and decrease to low dose when patient afebrile |
| \| \| **Follow Up Test** \| **At Presentation** \| **Follow up Frequency** \| **Utility of Test** \| \| --- \| --- \| --- \| --- \| \| CBC \| Y(Mandatory) \| Every other day till discharge \| Lymphopenia, neutrophilia, Thrombocytosis \| \| CRP \| Y(M) \| 48 Hrs. After admission, then depending on trend \| Rising trends helps monitor the disease and treatment \| \| ESR \| Y(Optional) \| 48 Hrs. After admission, then depending on trend \| Rising trends helps monitor the disease and treatment \| \| Ferritin \| Y(M) \| Depends on Clinical condition \| Rising trends helps monitor the disease and treatment \| \| D-Dimer \| Y(M) \| Depends on Clinical condition \| Helps in choosing the anticoagulation \| \| AST, Sodium, BUN: Cr \| Y(M) \| Every 48 Hours  Before discharge \| Monitor treatment response.  End organ involvement \| \| Pro BNP \| Y(M) \| Optional, repeat to see the trend. \| Helps monitor myocardial injury. \| \| Trop-T \| Y(M) \| Optional, repeat to see the trend. \| Helps monitor myocardial injury. \| \| Echocardiogram \| Y(M) \| 48hours, before discharge and two weeks after discharge (if not available send for Echo after stabilization) \| Important in making diagnosis and follow up \| \| ECG \| Y(M) \| On clinical judgement \|  \| \| COVID-PCR \| Y(M) \| If first negative, to repeat between7-21 days of illness \| For establishment of Diagnosis \| \| COVID-Serology \| Y(M) \| Especially if PCR is negative.  Can be omitted due to resource limitation if PCR is positive \| For establishment of Diagnosis \|   **POST DISCHARGE FOLLOW UP** \| \| --- \| --- \| --- \| --- \| --- \| --- \| --- \| --- \| --- \| --- \| --- \| --- \| --- \| --- \| --- \| --- \| --- \| --- \| --- \| --- \| --- \| --- \| --- \| --- \| --- \| --- \| --- \| --- \| --- \| --- \| --- \| --- \| --- \| --- \| --- \| --- \| --- \| --- \| --- \| --- \| --- \| --- \| --- \| --- \| --- \| --- \| --- \| --- \| --- \| --- \| --- \| --- \| --- \| \| - All patients should be discharged home on ASA 5mg/kg/day unless contraindicated. - All patients should have follow-up in COVID-19 cardiology) 2 weeks post discharge for clinical evaluation, repeat echocardiogram, and management of steroid taper. • - Additional follow-up depending on presenting symptoms and clinical indications \| \|  \| |
