# Supplementary material for: Multisystem inflammatory syndrome (MIS-C) in Pakistani children: A description of the phenotypes and comparison with historical cohorts of children with Kawasaki disease and myocarditis
Source: PLoS One. 2021 Jun 21;16(6):e0253625. doi: 10.1371/journal.pone.0253625 (PMC8216534; doi:10.1371/journal.pone.0253625)
Supplement: S2 Appendix — Table 1. Clinical and laboratory features and outcome of patients presenting with MIS-C according to WHO case definition with or without history of contact. Table 2. Normal range of different laboratory values. Table 3. Expired Patients (n = 6) during the study period. Table 4. Multinomial model to predict phenotype of disease from clinical and laboratory data. (DOCX) [file pone.0253625.s002.docx]

**S2 APPENDIX**

**Table 1- Clinical and laboratory features and outcome of patients presenting with MIS-C according to WHO case definition with or without history of contact:**

| **Characteristics** | **With Contact History** | **Without**  **Contact History** | **Total** | **P-value** |
| --- | --- | --- | --- | --- |
|  | **N=21** | **N=9** | **N=30** |  |
| **Demographic Characteristics** |  |  |  |  |
| **Age in Month, Median (IQR)^α^** | 24(12-90) | 8 (4-48) | 24 (9-60) | 1.000 |
| **Age in Month, sub-group n (%)** |  |  |  | 1.000 |
| 1-11 Month | 3 (14%) | 5 (56%) | 8 (27%) |  |
| 12-23 Month | 4 (19%) | 1 (11%) | 5 (17%) |  |
| 24-59 Month | 6 (29%) | 1 (11%) | 7 (23%) |  |
| ≥ 60 Month | 8 (38%) | 2 (22%) | 10 (33%) |  |
| **Gender** |  |  |  | 1.000 |
| Male | 15 (71%) | 8 (89%) | 23 (77%) |  |
| Female | 6 (29%) | 1 (11%) | 7 (23%) |  |
| **Symptoms on Admission** |  |  |  |  |
| Fever | 21 (100%) | 9 (100%) | 30 (100%) |  |
| Length of Fever in Days | 4 (3-5) | 5 (4-5) | 4 (3-5) | 1.000 |
| Vomiting | 10 (48%) | 3 (33%) | 13 (43%) | 1.000 |
| Heart Failure | 11 (52%) | 3 (33%) | 14 (47%) | 1.000 |
| Hypotension | 9 (43%) | 2 (22%) | 11 (37%) | 1.000 |
| Diarrhea | 3 (14%) | 5 (56%) | 8 (27%) | 0.285 |
| Rash | 11 (52%) | 5 (56%) | 16 (53%) | 1.000 |
| **Clinical and laboratory Features** |  |  |  |  |
| **Clinical Diagnosis** |  |  |  | 1.000 |
| KD^β^ like illness | 8 (38%) | 4 (44%) | 12 (40%) |  |
| Non KD like illness with depressed cardiac function | 8 (38%) | 2 (22%) | 10 (33%) |  |
| Non KD like illness with TSS ^π^ | 5 (24%) | 3 (33%) | 8 (27%) |  |
| **COVID-19PCR Test Done** | 18(85.7) | 8(88.8%) | 26(86.6) | 1.000 |
| Positive | 9 (50%) | 5 (63%) | 14 (54%) | 1.000 |
| Negative | 9(50%) | 3(38%) | 12(46%) |  |
| **COVID-19antibody Test Done** | 5(23%) | 2(22%) | 7(23%) | 1.000 |
| Positive | 5(100%) | 2(100%) | 7(100%) | 0.345 |
| Negative | 0(0%) | 0(0%) | 0(%) |  |
| Neutrophils, Median (IQR) | 79 (71-85) | 72 (57-75) | 75 (66-85) | 1.000 |
| Lymphocytes, Median (IQR) | 14 (10-20) | 20 (17-27) | 18 (11-24) | 1.000 |
| Platelets Count, Median (IQR) | 230 (160-317) | 245 (98-331) | 230 (140-330) | 1.000 |
| CRP^∞^, Median (IQR) | 21.00 (3.05-87.44) | 41.75 (15.25-67.91) | 31.00 (3.05-84.85) | 1.000 |
| Pro-BNP^€^, Median (IQR) | 12536 (2214-25245) | 7509 (1230-11932) | 11862 (2063-25000) | 1.000 |
| TROP, Median (IQR) | 0.500 (0.132-0.900) | 0.200 (0.050-0.612) | 0.452 (0.083-0.900) | 1.000 |
| D-Dimer on Admission, Median (IQR) | 3.30 (0.50-30.00) | 14.00 (10.00-18.00) | 6.80 (1.00-24.90) | 1.000 |
| Ferritin, Median (IQR) | 383 (250-791) | 1000 (554-1527) | 587 (285-1035) | 1.000 |
| LDH^µ^, Median (IQR) | 596 (372-1697) | 776 (406-849) | 617 (406-1460) | 1.000 |
| **Coronary Involvement n (%)** | 5 (24%) | 5 (56%) | 10 (33%) | 1.000 |
| **Type of Coronary Involvement, n (%)** |  |  |  | 1.000 |
| Dilation | 2 (40%) | 0 (0%) | 2 (20%) |  |
| Aneurysm | 1 (20%) | 1 (20%) | 2 (20%) |  |
| Echo bright | 2 (40%) | 4 (80%) | 6 (60%) |  |
| **Treatment** |  |  |  |  |
| ICU ^∞^Admission | 18 (86%) | 8 (89%) | 26 (87%) | 1.000 |
| Steroids | 17 (81%) | 7 (78%) | 24 (80%) | 1.000 |
| IVIG^£^ | 9 (45%) | 3 (33%) | 12 (41%) | 1.000 |
| Anticoagulation | 14 (67%) | 9 (100%) | 23 (77%) | 0.720 |
| **Outcome** |  |  |  | 1.000 |
| Survived | 16 (76%) | 8 (89%) | 24 (80%) |  |
| Expired | 5 (24%) | 1 (11%) | 6 (20%) |  |

^∞^CRP: C-Reactive Protein, ^β^ KD: Kawasaki like disease, ^∞^ICU: Intensive care unit, ^α^IQR: Interquartile rate, ^£^ IVIG: Intravenous immunoglobulin,

^€^ Pro- BNP: brain natriuretic peptide, ^π^TSS: Toxic Shock syndrome.

**Table 2: Normal range of different laboratory values:**

| **Laboratory Investigation** | **Normal Range** |
| --- | --- |
| Total Leukocyte Count | 4.8-11.3 x10^9^/L |
| Neutrophils | 34.9-76.2 % |
| Lymphocytes | 17.5-45 % |
| Platelets | 154-433 x10^9^/L |
| C-Reactive Protein (CRP) | 0-10 mg/L |
| Brain-Natriuretic Peptide (BNP) | <100 pg/mL |
| D-Dimer | < 250 ng/mL |
| Prothrombin Time (PT) | 11-13.5 s |
| Serum Ferritin | 20-250 ng/mL for adult males  10-120 ng/mL for adult females, 18 to 39 years  12-263 ng/mL for females, 40 years and older  25-200 ng/mL for newborns  200-600 ng/mL for 1 month old  50-200 ng/mL for 2-5 months old  7-140 ng/mL for children 6 months to 15 years |
| Lactic acid dehydrogenase (LDH) | 140-280 IU/L |
| Left ventricle ejection Fraction (LVEF) | 50-70% |

**Table-3: Expired Patients (n=6) during the study period:**

| **Case #** | **Age**  **(Months)** | **Presenting**  **Symptoms** | **Time**  **of Death since arrival** | **Spectrum of Disease**  **(MIS-C)** | **History of**  **Contact** | **PCR/Serology** **Result** | **Treatment** **Given** | **Laboratory Findings** | **Chest X Ray** | **Echocardiography**  **Findings** |
| --- | --- | --- | --- | --- | --- | --- | --- | --- | --- | --- |
| **1** | 24 | Fever, Hypotension, poor perfusion, no  Urine out put | 4 hours | MISC-Viral Myocarditis. | Positive | PCR Positive | Inotropes only. | TLC=1 (×10^9^/L), Lymphocytes=10%Neutrophils=87%,  Platelets 230, Troponin I, Pro BNP= Not done | Cardiomegaly and  Pulmonary edema | LVEF=20%, Moderate  MR Mild Pleural and  Pericardial effusion |
| **2** | 12 | Fever for 3 days,  Seizures, Generalized maculopapular Rash,  Hypotension, Poor perfusion, decreased urinary output | 6-8 hours | MISC-Viral Myocarditis | Positive | PCR Not sent | Inotropes | TLC=9 (×10^9^/L), Lymphocytes=18%  Neutrophils=72%, Platelets=225(×10^9^/L)  Trop I=1.2ng/L, Pro BNP= 23750pg/ml, S. Ferritin=620ug/L  ALT=265 IU/L | Cardiomegaly  Pulmonary edema | LVEF=30%  Moderate MR  Mild Pleural and  Pericardial effusion |
| **3** | 60 | Fever for 5 days, with Diarrhea and  Vomiting.  Irritability  Hypotension and decreased perfusion | 3 hours | MISC-Viral Myocarditis | Positive | Not sent | Inotropes | TLC=10 (×10^9^/L)  Lymphocytes=15%  Neutrophils=79%  Platelets=415 (×10^9^/L)  ESR=77mm/hour, CRP=46mg/L  Trop I=0.9ng/L, Pro BNP= 2385pg/ml,S. Ferritin=198 ug/L, ALT=113 IU/L | Cardiomegaly  Pulmonary edema | LVEF=30%  MILD MR  Mild Pericardial effusion |
| 4 | 8 | Fever for 2 days, preceded by vomiting, diarrhea, hypotension and decreased urinary output | 3 hours | MISC-Viral Myocarditis | Negative | Not sent | Inotropes  Aspirin  Steroids | TLC=8 (×10^9^/L), Lymphocytes=11%, Neutrophils=80%  Platelets=350(×10^9^/L)  CRP=49mg/L  Trop I=1.2 ng/L  Pro BNP= 2385 pg/ml  S. Ferritin=1000 ug/L  ALT=142 IU/L | Cardiomegaly  Pulmonary Edema | Echo not done |
| 5 | 36 | Fever for 5 days,  Rash, Fits,  Decreased  Urinary output.  Decreased  perfusion. | 24 hours | MIS-C Toxic Shock Syndrome(TSS) | Positive | PCR-Positive | Fluids  Steroids  Inotropes  Extubated within 24 hours | TLC=11 (×10^9^/L)  Lymphocytes=10%  Neutrophils=86%  Platelets=212(×10^9^/L)  CRP=20mg/L  Trop I=0.5 ng/L  Pro BNP= 255 pg/ml  S. Ferritin=750 ug/L ALT=4186 IU/L  Blood CS  Negative | Normal cardiac shadow, no  Pulmonary edema | LVEF=55% MILD MR  Repeat Echo showed LVEF-60 %.  No PE |
| **6** | 10 | Fever for 3days,  Vomiting diarrhea hypotension and decreased perfusion. | 3 | MISC-Viral Myocarditis. | Positive | Positive | Inotropes  Steroids | TLC=08(×10^9^/L)  Lymphocytes=11%  Neutrophils=86%  Platelets=139(×10^9^/L)  CRP=20mg/L  Trop I=1.7 pg/ml  Pro BNP= 30206 ng/L  S. Ferritin=383 ug/L  LDH 1460 IU/L | Cardiomegaly  Pulmonary | LVEF=30%  No MR  No PE. |

^µ^ALT: Alanine transaminase, ^∞^CRP: C-Reactive Protein, ^β^ LVEF: Left Ventricle Ejection Fraction, ^π^MR: Mitral Regurgitation, ^€^ Pro- BNP: brain natriuretic peptide,

^α^ TLC: Total Leucocyte count.

**Table 4: Multinomial model to predict phenotype of disease from clinical and laboratory data:**

| **Predictors** | **KD MIS-C** | | **VM-MIS-C** | |
| --- | --- | --- | --- | --- |
|  | **RR (CI 95%)** | **P-value** | **RR (CI 95%)** | **P-value** |
| Age in Month | 0.99(0.97 - 1.00) | 0.26 | 0.98(0.97 - 1.00) | 0.2 |
| Gender: Male² | 0.27(0.02 - 3.66) | 0.32 | 2(0.26 - 15.38) | 0.5 |
| Leucocyte | 0.93(0.82 - 1.05) | 0.26 | 0.92(0.81 - 1.06) | 0.27 |
| Neutrophils | 1(0.94 - 1.08) | 0.8 | 0.98(0.91 - 1.04) | 0.56 |
| Lymphocytes | 0.96(0.87 - 1.05) | 0.38 | 1.01(0.94 - 1.08) | 0.68 |
| Platelets Count | 1.01( 1.00 - 1.02) | 0.017 | 1(0.99 - 1.02) | 0.142 |
| CRP ^∞^ | 1.00(0.99 - 1.02) | 0.27 | 0.98(0.94 - 1.01) | 0.23 |
| Pro-BNP, ^€^ | 0.99(0.99 - 1.00) | 0.17 | 1(0.99 - 1.00) | 0.4 |
| Troponin | 1.01(0.56 - 1.81) | 0.97 | 1.05(0.60 -1.83) | 0.85 |
| D-Dimer | 1.01(0.92 - 1.10) | 0.76 | 1.03(0.90 - 1.17) | 0.64 |
| Prothrombin Time. | 0.97(0.91 - 1.04) | 0.52 | 1.03(0.97 - 1.09) | 0.3 |
| Ferritin | 1(0.99 - 1.00) | 0.43 | 0.99(0.99 - 1.00) | 0.36 |
| Lactic acid dehydrogenase | 0.99(0.99 - 1.00) | 0.64 | 0.99(0.99 - 1.00) | 0.77 |
| ALT^µ^ | 0.99(0.99 - 1.00) | 0.26 | 0.99(0.99 - 1.00) | 0.35 |
| Admission ICU ^∞/^ | 1.4(0.10-18.6) | 0.799 | 0.77(0.04 - 14.74) | 0.86 |
| IVIG ^£^ | 0.06(0.007 - 0.60) | 0.16 | ---- | --- |
| IVIG and Steroids | 0.46(0.06 - 3.344) | 0.44 | ---- | --- |
| Anti-coagulation | 8.97-08 | 0.99 | 1.11(0.16 - 7.50) |  |
| MR^π^ | 4.5(.57 35.51) | 0.15 | 0.12(0.01- 1.52) | 0.1 |
| Pericardial Effusion | 1.31E+07 | 0.99 | 0.17(0.02 - 1.43) | 0.1 |
| LVEF ^β^ Admission | 1.04(0.98 - 1.10) | 0.17 | 1(0.94 - 1.06) | 0.9 |
| Coronary Involvement | 0.42(0.06- 2.68) | 0.36 | --- | - |
| Echo-bright Coronary | 1.49(0.22 -10.21) | 0.67 | --- | - |
| Follow up | --- |  | 8.00(0.59 -106.97) | 0.11 |
| Outcome | --- |  | 6.99(0.61 - 79.80) | 0.11 |
| Base category is MIS-C TSS | |  |  |  |
| ²compare with female |  |  |  |  |

^µ^ALT: Alanine transaminase, ^∞^CRP: C-Reactive Protein, ^∞/^ ICU: Intensive care unit, ^£^ IVIG: Intravenous immunoglobulin, ^β^ LVEF: Left Ventricle Ejection Fraction,

^π^MR: Mitral Regurgitation, ^€^ Pro- BNP: brain natriuretic peptide.
